# The discovery of fire by humans: a long and convoluted process

**DOI:** 10.1098/rstb.2015.0164

**Published:** 2016-06-05

**Authors:** J. A. J. Gowlett

**Affiliations:** Archaeology, Classics and Egyptology, School of Histories, Language and Cultures, University of Liverpool, 12-14 Abercromby Square, Liverpool L69 7WZ, UK

**Keywords:** fire, human evolution, archaeology, palaeoanthropology

## Abstract

Numbers of animal species react to the natural phenomenon of fire, but only humans have learnt to control it and to make it at will. Natural fires caused overwhelmingly by lightning are highly evident on many landscapes. Birds such as hawks, and some other predators, are alert to opportunities to catch animals including invertebrates disturbed by such fires and similar benefits are likely to underlie the first human involvements with fires. Early hominins would undoubtedly have been aware of such fires, as are savanna chimpanzees in the present. Rather than as an event, the discovery of fire use may be seen as a set of processes happening over the long term. Eventually, fire became embedded in human behaviour, so that it is involved in almost all advanced technologies. Fire has also influenced human biology, assisting in providing the high-quality diet which has fuelled the increase in brain size through the Pleistocene. Direct evidence of early fire in archaeology remains rare, but from 1.5 Ma onward surprising numbers of sites preserve some evidence of burnt material. By the Middle Pleistocene, recognizable hearths demonstrate a social and economic focus on many sites. The evidence of archaeological sites has to be evaluated against postulates of biological models such as the ‘cooking hypothesis' or the ‘social brain’, and questions of social cooperation and the origins of language. Although much remains to be worked out, it is plain that fire control has had a major impact in the course of human evolution.

This article is part of the themed issue ‘The interaction of fire and mankind’.

## 1. Introduction

Fire is universally accepted as important to human life, with myriad expressions and uses in the modern world [1–7]. It was regarded by Darwin as the greatest discovery made by humanity, excepting only language [8]. Although open fire tends to be built out of Western technology, it persists in many forms as hidden fire, as in the internal combustion engine. Fire has underpinned the development of all modern technologies—from ceramics, to metal working, to the nuclear industry.

**Figure 1.**
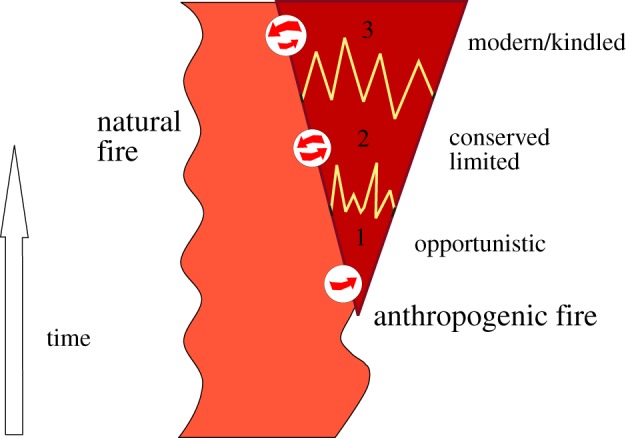
A putative general outline for the development of human fire use, showing its emergence from and interchanges with natural wildfire. All boundaries can be regarded as highly fluid: it is highly likely that there are different fire histories on different latitudes and continents.

This paper starts with the view that such human fire use is an offshoot or outgrowth of far older natural fire regimes [9–15] (figure 1), and it aims to address two main issues: when and how humans came to be engaged with fire; and what are the main long-term impacts that their fire use has had on the natural environment? In the first place, large numbers of lightning strikes would have made fire evident to early humans in the form of bush fires, even aside from other rarer forms of natural ignition such as volcanic activity [16]. Archaeology and anthropology have often treated fire as a technological ‘add on’ or invention, but fire awareness must inevitably go back to very early times because of the high visibility of natural fires. The early encounters have been followed by an intensification of use which has had profound impacts on human culture and even biology [17]. Fire has played a major role in transforming human diet [18], and apart from its major impact on environments, it has become socially embedded, even to the point of having religious significance and being incorporated in ritual [1,19,20].

The evolution of the primates from about 70 Ma [21,22] provides the ultimate background for encounters with fires in landscapes. Their development is largely owed to the ‘angiosperm revolution’ [10,11,23], in which flowering and fruiting trees provided niches for tree-living insectivores and especially frugivores as well as folivores. By 35 Ma ape-like and monkey-like primates had appeared. For more than 20 Myr, recognizable apes were widespread as denizens of forests [24]. Although lightning can on occasion cause tropical forest fires, in general they would not have been considerably exposed to fire in these moist densely vegetated environments [25,26]. Within the last 10 Myr, however, pivotal climate and vegetation changes led to new habitats and new adaptations across the Old World, and in that context the evolution of the hominids [27]. Along with C4 plants such as grasses, mammal groups such as horses were able to disperse through Africa [23,28,29], and tropical forest was replaced over large areas by wooded, bushy or more open habitats.

**Figure 2.**
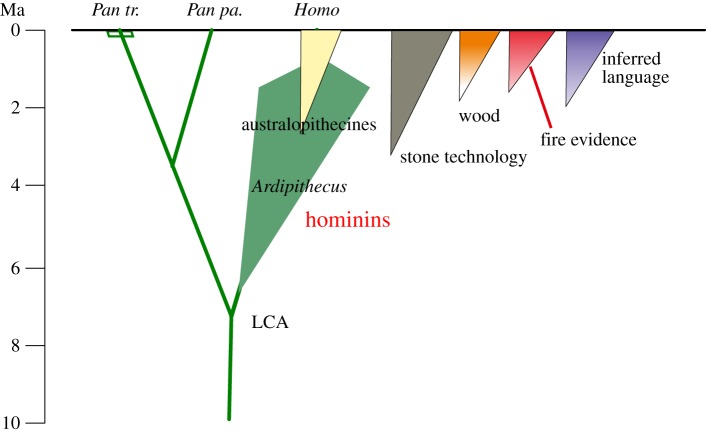
The emergence of the hominins: chart indicating the relationships with chimpanzees and bonobos (*Pan troglodytes* and *Pan paniscus*), and the staging of the major hominin adaptations and culture. Of these, hard technology, fire and language can be seen as ‘the big three’, deeply connected in the end and perhaps at earlier stages. LCA, last common ancestor of hominins and *Pan*.

The earliest hominins probably diverged from apes around 6–8 Ma [30], and their evolution can be seen as a response to these changes—apes who, as the final part of a Miocene ape radiation, adapted to new wooded environments [31]. Rather than apes who came down from the trees, as traditionally seen, our ancestors were the bush country apes, and as such, through the last 3 Myr especially, some of them became exposed to more open habitats where natural fire was much more prevalent and obvious. The period 6–3 Ma, the first half of this evolution—the time of *Ardipithecus* and its relatives [32]—involved adaptations of bipedalism and life in wooded environments, accompanied by features such as reduction of jaws and teeth and lengthening of the thumb [31–33]. The second half indicates, for *Homo* lineages at least, a new complex of adaptation committed to long ranging, open environments, meat eating and other new foods [34–36]. In this context, encounters with fire must have become far more frequent and significant (figure 2).

A series of recent finds has given us a changed deep picture of the hominins, showing that their engagement with technology reaches back as much as 50% of the way to hominin origins. Stone tool finds from Lomekwi 3 at West Turkana in northern Kenya push back the hard record of technology from 2.6 to 3.3 Ma [37]. Such finds are important, because they almost certainly indicate a knowledge of working wood as well as stone, and hence of properties of friction and heat. At the same time, new finds from northern Ethiopia set the origins of our own genus, *Homo*, as early as 2.8 Ma [38]. These discoveries square with others that indicate a dispersal of hominins across the Old World far earlier than was expected a few years ago—dates of 1.8 Ma in Georgia and eastern Syria, 1.7 Ma in northern China and more than 1.5 Ma in Java are strong indicators that the actual dispersal goes back further, perhaps more than 2 Myr [39–43]. It has the effect of putting hominids as far north as 40°N, at this early date, indicating that unlike the great majority of primates they had evolved means to cope with summer–winter seasonality.

Altogether, a more complex picture of early *Homo* has emerged, with regional diversity, smaller brains than were expected, and coexistence with other hominins such as the robust australopithecines for at least 1.5 Myr. Stone tool transport distances show that these animals ranged over large territories which were often open in character [44,45]. Recent research has also given a broader picture of other primate behaviour. The sophistication of ape behaviour has been recognized, including their technology. In West Africa, Pruetz and LaDuke have shown the use of wood weapons by savanna chimpanzees, and their awareness of fire [46]. We must be alert then to possibilities that hominins could have been interacting with fire in simple ways from an early date [47].

## 2. Origins of interactions with fire

Archaeological research has tended to concentrate narrowly on the presence or the absence of hearths, largely because of its own focus on living sites [48]. In broader evolutionary scenarios, it is evident that we have to consider at least three distinct but potentially intergrading forms of fire use: first, fire foraging for resources across landscapes; second, social/domestic hearth fire, for protection and cooking; and third, fires used as tools in technological process, e.g. for firing pottery.

Modern fire use is highly complex, but its origins are likely to have been simple: a common biological rationale is that there is one main selective pressure for a new development of this kind [49]. For humans, fire became important for many reasons, including cooking, protection and warmth, but most of these presuppose some degree of control. Fire foraging, in contrast, demands only an attraction towards fires, in the hope of benefitting from additional resources [17,49]. For hominins, benefits could include retrieval of birds eggs, rodents, lizards and other small animals, as well as of invertebrates. Although fire does not create such resources, it renders them far more visible, and chance cooking might well improve their digestibility.

Support for the primacy of foraging comes from the animal world. Although only humans have full mastery of fire, and it has been said that there is no analogue, there are occasional instances, largely anecdotal, of mammalian predators such as cheetahs positioning themselves to spring on prey fleeing fires. Bird ‘fire followers' are much better recorded. They amount to many species across continents [50]. They show the availability of resources, the potential selective advantage, and by inference that this kind of fire harvesting would be within the cognitive capabilities of early hominins [51].

From simple interactions, the challenge to hominins would be to stretch fire, both in space and time, to enhance its utility. In Alaska—a reasonable proxy for parts of ice age Europe—the fires burn largely from June to September. Thus, fire would not be available through the cold parts of the year, unless it could be maintained effectively. In Africa, the challenge might be to maintain fires through the wet seasons. Any such efforts, indeed almost all fire management, pushes towards a division of labour. Slow-burning materials such as animal dung or plant material tapers need to be selected and guarded, while other subsistence activities go on.

Without doubt, natural fire was available on the landscapes inhabited by hominins. Of the millions of lightning strikes that are recorded each year [16], many lead to bush and forest fires, especially at the start of a rainy season: then lightning from the first thunder storms often strikes when much of the vegetation remains dry [52–58]. Most of the instances of relevance are in forest, woodland and savanna, but the fire regimes operate surprisingly far north. Farukh & Hayasaka [59] give the example of Alaska, where up to 100 fires are burning on a given day in the summer season, and important for hominins, they have an average duration of more than 20 days.

## 3. Sampling the record of early fire

In total, the early archaeological record documents many thousands of events of hominin activity, but the chances of fire being preserved are exceptionally small. This is in part because of its ‘disappearing act’—there remain scant traces of burning, rather than the fire itself [5]—and partly because of the overall low density of sampling. As stone tools endure far better, their record is full enough to give some insights into sampling. When the Lomekwi 3 site at West Turkana in Kenya was published it took the record back from 2.6 to 3.3 Ma [37]—amounting to one sampling of the ‘new’ 700 000 years. If hominins had actually made tools (say) 10 times a year, then with a population of (say) 10 000, current sampling would give a 1 in 70 billion chance of recovery. If that seems excessively hypothetical, we can come forward to the period 2–1 Ma: there are some hundreds of archaeological occurrences in total, but currently a maximum of five preserving evidence of burning (mentioned below). Fire is therefore about 10–100 times less likely to feature than hard artefacts. In that light, it seems remarkable that overall we do have so much fire in the record.

## 4. Major biological models

Fire foraging would lead inevitably to consumption of foods cooked accidentally, including the ‘roots made digestible’ mentioned by Darwin. The basis of the cooking hypothesis as set out by Wrangham and colleagues is that hominins living in more open environments would be unable to feed through the year from the fruit and herb resources which sustain apes in tropical forest. They would need to adopt other foods, particularly during dry seasons [34]. Extending their use of meat and particularly of carbohydrates in the form of roots and tubers would be necessary for filling this gap [35,36,60]. Large teeth—megadonty—hint at dietary stress in the period before 3 Ma, and isotopic studies at the incorporation of new foods such as grasses and sedges [61,62]. From as early as 2.6 Ma, increased meat eating is well attested by archaeological sites that link stone tools and cut-marked bones [44,63].

But the new foods are hard to digest. Cooking greatly increases their digestibility: in the view of Wrangham and colleagues, this would have come with *Homo erectus* at about 1.7 Ma [64–66]. Part of the evidence advanced is that a modern human body plan emerges at this time, with features including lengthened hindlimbs [67], and reduction of sexual dimorphism [68]. In particular, the teeth of *Homo erectus* are reduced in size, sometimes as much as those of modern humans making allowance for body size ([68], cf. [69]).

In a sense, the cooking hypothesis is proved, in that all modern humans need cooked food [66]: the question therefore is whether the hypothesis can be locked into a fixed position in the past, a rapid switch of adaptation. This is far harder to demonstrate, given our inadequate picture of early hominin species variation, and the variety of environments which they inhabited. As a working hypothesis, however, this set of ideas brings to life the problems that early hominins were working against in terms of processing foods, and living alongside large predators.

A striking increase in human brain size is also one of the major developments in *Homo*. It has risen from an average *ca* 600 to 1300 cc in the course of the Pleistocene [70,71]. As a larger brain is costly in energy, it needs explanation. The social brain hypothesis aims to explain the phenomenon in terms of increases in group size and pressures towards social cognition [72–74]. High-quality diets are a necessity of fuelling the larger brain, from early times and especially from half a million years ago [68,72,75]. Social brain calculations suggest rapid change at this stage, and a link with language origins [71,76].

These hypotheses can be seen as promoting ‘step changes' in hominin evolution—but the genetic comparisons now possible from whole genome studies indicate a steady progression of many complex changes, rather than any Rubicon [77].

## 5. Recognizing fire in the record

Fire on landscape is of deep interest, but it is practically impossible to distinguish between wildfires and similar fires that may have been started by humans. Some of our best clues as to how this might be done come from Australia. In a modern instance, the Martu people of the western desert only gave up their traditional fire stick farming methods in the 1960s. The change led to a great rise in the size of individual fires [78,79]. Through the systematic use of small fires the aborigines had habitually managed small mammal communities in a way that appears to enhance resources [80]; other hunter–gatherer studies imply also a concern for enhancing vegetation [54].

More generally, archaeological methodology has to focus on the restricted domains of sites where there has been notable human activity—possible home bases. The idea of the home base has been much debated [80–82], but dense concentrations of stone tools as much as 2.5 Ma show that hominins remained in one place long enough or frequently enough that overnight stays were likely [83,84]—and if fire was in use it was likely to be employed on some of these, although the chances of preservation are very slight.

On occasion archaeology is capable of recognizing artefact evidence of fire beyond all doubt. One case is a preserved wooden fire ‘hearth’ from Guitarrero Cave in Peru, directly dated by radiocarbon to around 2000 years BP; cord and dowels from the site date to *ca* 10 ka [85,86]. The sockets where the fire drill was inserted are plainly visible.

Another is lumps of pitch preserved from a Neanderthal site at Königsaue in the foothills of the Harz Mountains in Germany [87]. Pitch, probably used as a fixative in hafting, can be made from tree bark only by maintaining high temperatures in a controlled fire for several hours. This can be regarded as almost the ideal case of fire documentation, since one piece of pitch retained a human fingerprint, and direct radiocarbon dating gave an age of *ca* 48 000 BP, on the limits of the technique, and compatible with a geological age of approximately 80 000 years. The use of gypsum plaster for hafting in the Middle East also implies the use of fire [88].

Occasionally, elsewhere, wooden artefacts may be part burnt or burnt. At Kalambo Falls in Zambia burnt wooden artefacts were found on Acheulean sites dating to *ca* 0.5 Ma [89,90]. At Beeches Pit, mentioned below, a refitting flint artefact set included two burnt specimens in the set of 27, a circumstance not readily consistent with natural fire [91,92].

Such examples emphasize the importance of context, and the point that an organized methodology is necessary for fire enquiries. In archaeology, a first general treatment was provided by Bellomo in the 1990s [93,94]; subsequently, micromorphological studies of sediments, magnetic methods—including magnetic susceptibility and palaeomagnetic techniques—and thermoluminescence measurements have all proved highly useful [95,96].

No technique on its own completely addresses the problems of enquiry. The strength of micromorphology is obviously its ability to look at the small scale. The scaling up to provide evidence of specific human actions is therefore more likely to come from archaeology; but multiple techniques are necessary for any full picture. Thermoluminescence and magnetic methods can provide estimates of critical factors such as temperatures and duration of burning [97].

## 6. Fire origins in the archaeological record

**Figure 3.**
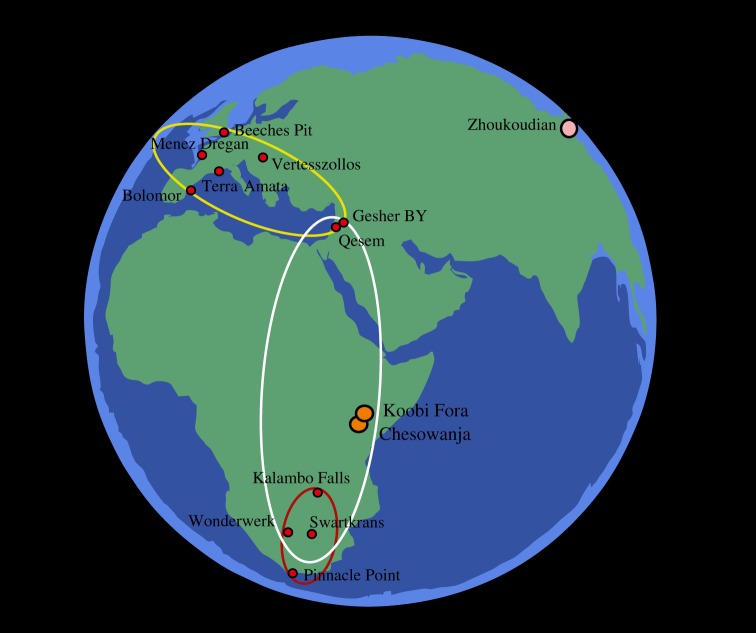
Some major Pleistocene sites with traces of fire. Following earliest traces at Koobi Fora and Chesowanja, *ca* 1.5 Ma, the ovoids indicate the biases in representation: centre, occurences *ca* 0.7–1.0 Ma; Europe/Mediterranean, 400 000 years onward; southern African: *ca* 0.5 Ma onwards. In the Far East, Zhoukoudian (*ca* 0.7 Ma) is followed by other sites with fire traces.

The two earliest sites are in Kenya: FxJj20 at East Turkana, and site GnJi 1/6E in the Chemoigut Formation at Chesowanja near Lake Baringo (figure 3). These are both open sites. According to the original publications, FxJj20 preserves burned sediments and some heat-altered stone tools [98,99]. The site remains a strong candidate for early fire use and is currently under complete reinvestigation (S. Hlubik 2015, personal communication). Chesowanja preserves somewhat similar information, but the burnt material at the centre of the site consists not of a burnt patch, but of a few large clasts of baked clay [100,101]. The possibility that they could come from an adjacent (but lost) natural burning feature is difficult to exclude on present evidence, although the clasts are directly associated with numerous stone tools and faunal remains. A site at Gadeb in Ethiopia is also of similar age [102].

Several sites then range through the period approximately 1.0–0.5 Ma. They include the very different cave sites of Swartkrans and Wonderwerk in southern Africa, and the open site of Kalambo Falls in Zambia (mentioned above). At Swartkrans, in Member 3, described as a roofed gully, fragments of burnt bone were found in 17 excavation squares, arguing against their creation by occasional savanna fires sweeping up to the site [103–107]. They include several specimens also showing cutmarks from butchery. At Wonderwerk Cave, micromorphology studies in stratum 10, dating to approximately 1 Ma, indicate that quantities of grass and other vegetation were introduced far into the cave and became burnt along with bone preserved as microscopic fragments [108,109]. The important site of Gesher Benot Ya'aqov in Israel preserves burnt materials at numerous levels in a 30 m sequence dating to *ca* 700 000 years [110–112]. Charcoal was identified at 10 levels, and burnt wood at 4. Most specifically, burnt flint microartefacts were found in clusters which mark out ‘phantom hearth’ areas [110,112]. Macroscopic burnt flints and burnt pebbles have also been found, for example, 24 in total from the layer I1–6 L-7 [112].

Zhoukoudian near Beijing in China has been known for more than 80 years as a fire site [113,114]. Critiques have been made of its context, and on the nature of the ‘burnt’ material [115–118], much of which resulted from other natural processes. Nonetheless, the site is a record of the activities of *Homo erectus* in the period 0.4–0.7 Ma, with more than 100 000 artefacts, and preserving burnt bone [117,119,120]. The repeated associations argue for controlled fire [120].

From around 400 000 years ago, traces of fire become much more numerous on many sites, including numbers in Europe and the Middle East as well as Africa and Asia [80,121,122]. Qesem in Israel preserves a large hearth maintained over a period [123,124]; fire traces also appear regularly at nearby Tabun Cave at about the same time [125]. In northwest Europe, Beeches Pit, a 400 000 year old interglacial site in eastern England, has various traces of fire, suggesting that large hearths were maintained by the side of a creek. The traces include burnt bone, shells, combustion features, and most particularly the evidence of a refitting set of flint artefacts [91,92,122]. Of 27 flakes discarded in the process of shaping an intended handaxe, only two became heated and reddened, indicating highly localized burning.

Despite the increasing numbers of fire sites, their *relative* scarcity is still notable [126], as is the fact that some very major sites in Europe are totally lacking in fire evidence. These include lower levels at the Caune d'Arago at Tautavel in southern France, where among more than half a million finds of flints and bone there are no burnt traces older than 400 000 years [121]. At a later date, too, there are significant gaps in the fire representation in Mousterian sites [127]. By contrast, at approximately 300 000 years ago, Vertesszollos in Hungary, Terra Amata and Menez Dregan in France and Bolomor in Spain show frequent evidence of fire [121,128–131], continued in Spain on later Neanderthal sites such as Abric Romani [132].

**Figure 4.**
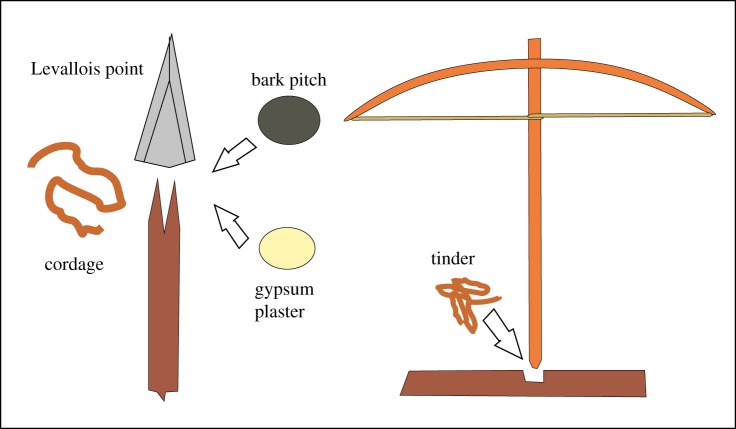
Hafting of a Levallois point: the implicit connections with fire. Hafting of Levallois may occur as early as 500 000 years ago [133–135]. Two glues in use by 50–100 ka require fire for preparation; twine, implied to be in use by 120 ka [123] is a requisite for working a fire drill. Hafting and the use of a fire drill involve a similar conceptual mastery of bringing together two components via a vital intermediary—fixative in the one and kindling in the other.

It has been argued a number of times that fire management may have improved markedly around 400 000 years ago [81,121–123,126]. The Levallois technique of stone working originates around the same period, and gives strong indications of the beginnings of hafting [133–135] (figure 4). This is also implied at two German sites, notably Schöningen, where short wooden staves are preserved with deep notches in the ends [136]. Effective hafted systems require glue or twine—it may be highly significant that two of the main glues require heat treatment for their production [87,88].

**Figure 5.**
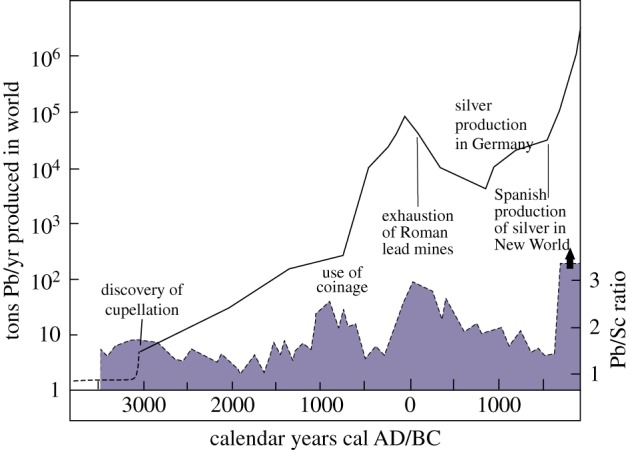
Full impact of fire use may come only when agricultural economies are followed by industrial ones. Here evidence of two records of metal exploitation demonstrates effects through the last 5000 years. The lead aerosol record of Arctic ice cores gives a dated index to production of lead and silver through the last 5000 years [139], and as such may provide an effective guide to the relative scales of burning of wood in industrial processes through that period, long before the atmospheric effects of fossil fuel burning are seen. Through the same period, lead/scandium ratios from a peat bog in the Basque country give indications of the local peak mining period which are sometimes also marked by signs of deforestation [140].

The question of ignition is an important one [127,137], but perhaps less crucial to effective fire use than often assumed. If hominins could not ignite fire, however, they would need to be able to maintain it robustly, and hence probably be reliant on a strong social network allowing its replacement [138]. They would need good knowledge of slow-burning materials, although field studies show that animal dung is useful in this respect. Ignition is often assumed to have required a cognitive advance. Yet the simplest kindling technique of rubbing a stick in a groove in a wooden ‘hearth’ requires no more than power and basic skill. It does not seem a more complex process than hafting, which it closely resembles in that two component parts require understanding and use of an intermediary: fixative in the one, and tinder in the other (figure 5).

By 120 000 years ago, pierced shell beads [141] indicate a knowledge of twine or leather cord, which would have been necessary for operating a fire drill. Before this date at Pinnacle Point in South Africa, stone was being warmed to improve its working qualities [142]. Such finds are a further early indication of the use of fire in technological processes: with its need for fuelling and maintenance domestic fire becomes a firm stimulus towards division of labour, planning and focusing of attention [17].

From this point, fire use can be seen as almost universal, as it is among living modern humans (e.g. [143–145]). Even so, there are puzzles in the record, where fire is seemingly inexplicably absent (as in some parts of the record in Middle Palaeolithic France [127]), and it remains possible—balanced against the vicissitudes of sampling and preservation—that the costs and risks of using it sometimes outweighed the benefits.

## 7. The impact of fire

Over a long period, human interventions have grown to the point that in the modern world fires started by humans usually vastly outnumber those started by nature. Even so, in areas such as the Great Basin of the southwest USA, lightning-started fires still outnumber anthropogenic fires by a factor of 2 or 3 to 1 [146]. In general, however, longstanding natural fire regimes have been interrupted and superseded. Recent syntheses make plain the importance of knowing when that becomes true in terms of landscape, and it is evident that geographical, ecological, archaeological and anthropological studies can come together far more effectively (e.g. [147]). The issues are complex for three main reasons which have to be meshed with the studies of natural fire regimes [10,12,15,149–171]. First, the dispersal of modern humans is marked by different arrival times in different regions—of the order of 50–60 ka for Australia and 40 ka for Europe [172,173], and 10–20 ka for the Americas [174], far later again for New Zealand and the Pacific [175]. Second, the arrivals and recolonizations sometimes cut across the immense climate changes involved in the transition from the last glacial maximum to the Holocene. A third key factor is that hunting and gathering economies began to be replaced by agricultural and pastoralist economies from about 10 000 years ago [176]. Until then, populations were relatively low, of the order of 1 person km^–2^, but farming raised population densities by at least 10 or 100 times: the significance of this is that most major human impacts are likely to be relatively recent, occupying less than 0.5% of the Pleistocene.

Modern hunter–gatherers do however demonstrate that people in small numbers can have significant effects [78,79]. Humanly influenced regimes are found across the world of hunter–gatherers [54,78,79,177–181], but to varied and debated extents. Principal questions are how far back they go in time, and how great their influence was. For Africa, Archibald *et al.* [54] have argued for a potentially greater influence through the last approximately 100 000 years, as early modern human populations increased. The main archaeological evidence comes from the shaping of the African Middle Stone Age (MSA), including greater transport distances for artefacts, and the eventual dispersal out of Africa [182,183]. The other signs of complex fire management, mentioned above [87,88,142] also suggest the possibility that the landscape scale interventions may extend back to 100–200 000 years ago, if not further. A rare study based on elemental carbon in a deep sea core indicates an increase in fire at about 400 000 years ago [184], but in the view of its specific association with interglacial to glacial transitions, there may be no anthropogenic implications.

As has been seen, in many parts of the world first interventions by colonizing modern humans would occur only at more recent dates. Accordingly, local fire histories may have far greater validity than global ones, and the time differences in human occupation give scope to compare records, especially across the southern continents.

Within the last 20 000 years, there came major new fire interactions, the first associated with pottery, which appears to have originated in China [185,186]. From around 10 000 years ago, agriculture would potentially have widespread effects. Fixed Neolithic settlements, such as Çatalhöyök, would have required wide-ranging foraging for firewood [187], but there are indications in the Levant that woodland was sometimes managed [188]. Soon afterwards, from roughly 5000 years ago come the beginnings of metalworking, first copper and bronze, and then iron. Such interventions involve the raising of temperatures far above those of open fires—the development of a true pyrotechnology [189]. Lead aerosols from arctic ice cores provide an index of lead and silver production through the last 5000 years [139], and can perhaps also be used as a rough proxy for the scale of burning across the Northern Hemisphere through the last 5000 years. They are consonant with local records of mining evidence, e.g. from the Basque country [140], where there are signs of periodic deforestation. The main impact came from the time of the Roman Empire onwards (figure 5).

It remains to consider the impact of fire on human biology and sociality. The change in the genus *Homo* over 2 Myr has been remarkable. There are signs that a considerable part of this can be put down to the influence of fire. Particularly striking is that modern adult humans have an exceptionally long waking day, of 16 h or more, compared with 8 h in many mammal species [190,191]. Whereas other primates such as chimpanzees and gorillas rise with the dawn and go to sleep around sundown [192], humans have peak alertness in the early evening [13,193,194]. The several additional hours of wakefulness appear to have been made possible by fire and its ‘daylight extension’. The reasons appear to have been for social time (hence a probable link with language: [49,71,72,138,195]), as well as protection against predators. Changes in the size and proportion of stomach, small intestine and large intestine may be part of the same complex—owed to changed diet, necessary for sustained movement on the ground, and following the expensive tissue hypothesis a possible co-requisite of the large human brain [75].

It is probably not an exaggeration to say that there was also a re-organization of human sociality focused on fire and the hearth. Earlier mention was made of the needs for division of labour. Costs of fire can be high, too: the longer a settlement is inhabited, often the greater the distances covered in fuel-foraging. Such aspects can probably be related to the emergence of larger group sizes, these also entailing the active support of a post-mature generation—grandfathers and grandmothers [196,197]—and of children [20].

From all this, it is clear that fire has had both direct and indirect impacts. Apart from its effects on the environment and human sociality, its influence has reached in some way into the human psyche, expressed in religion, in ritual, in ceremony [198] and through ubiquitous myths about fire origins [149,199].

## 8. Conclusion

The deep importance of fire, and the longstanding nature of human interactions with it in the past, are both beyond doubt. The vanishing act of early fire ensures that it remains difficult to investigate, so that widely varying views remain both about its first take-up and subsequent use, but recently a changed perception has emerged. First, there is an increasing recognition of a need to move beyond simple ‘presence/absence’ judgements about archaeological hearths as an index for the ‘when’ of human fire use. Regular human–fire interactions could long precede fixed hearths in settlements. Second, an understanding is emerging that fire use is not a single technology or process, but that several scales of use, and probably several intensifying technologies, evolved over a long period, intertwined, and sometimes eventually became bound together.

In total, we know a good deal, if much remains to be found out of the ‘why and when?’. We know that our nearest relatives, the chimpanzees, are not intimidated by fire, but behave sensibly in relation to it; that humans were exposed to fire frequently from the time that they moved into open savanna environments more than 2 Ma; from isotopic evidence and changes in teeth, that their diet altered considerably around this time. We know that burning evidence occurs on numbers of archaeological sites from about 1.5 Ma onwards (there is evidence of actual hearths from around 0.7 to 0.4 Ma); that more elaborate technologies existed from around half a million years ago, and that these came to employ adhesives that require preparation by fire. We know that both early modern humans and Neanderthals had sophisticated fire technologies, at least some of the time. Despite the huge biases of disappearance and preservation, a new phase of early fire research is emerging in which interdisciplinary approaches offer the chance of addressing questions with increased success. In the grand sweep of human evolution, ‘intensification’ is a dominant theme in the practices and culture of *Homo*: fire use is entirely in step with other lines of evidence.

## 9. Meeting discussion

*N. Roberts* (University of Edinburgh). What information is available regarding the size of the groups that would congregate around and use fires across archaeological times and in different regions of the world? Is there a latitude dependence perhaps relating to the need to provide warmth?

*J. Gowlett*. It is an important question, but up to 400 000 years ago any information we have relates to site size and group size, rather than how many congregated around a hearth. From around 400 000–300 000 years ago when numbers of structured hearths can be seen, they appear to include both large and small in different contexts. Size may depend on immediate purpose and available fuel more than climate. Social factors are also likely to determine whether fires are communal, or specific to nuclear families. We have Late Pleistocene sites such as Meer in Belgium where there are numbers of hearths of different sizes in a small settlement.

*C. Roos* (Southern Methodist University, USA). I appreciate your recommendation that we look to non-human animal analogies for how our hominin ancestors may have seen fire as an opportunity. Do you think that opportunistic fire-margin hunting or scavenging might account for the evidence for increased meat consumption around the time of early encephalization (instead of the cooking hypothesis)?

*J. Gowlett*. The analogy with other animals might suggest that in the first instance early hominins would go to fires simply to take advantage of any additional opportunities of gaining prey, regardless of whether the resources were cooked. For example, fire may reveal a clutch of eggs—so much the better if it has baked them. For encephalization, new cranial finds are altering the figures rapidly, but at the moment it would seem that the average cranial capacity for early *Homo* at 1.8 Ma is 600–650 cc, 40–50% greater than for most apes and australopithecines—and yet this is earlier than Richard Wrangham's postulated date of 1.7 Ma for applying the cooking hypothesis. Perhaps the fire foraging is one important element, and the cooking hypothesis comes into play more strongly later, but other factors operate alongside both.
